# Prevalence of Burnout in Healthcare Workers of Tertiary-Care Hospitals during the COVID-19 Pandemic: A Cross-Sectional Survey from Two Central European Countries

**DOI:** 10.3390/ijerph20043720

**Published:** 2023-02-20

**Authors:** Ladislav Štěpánek, Marie Nakládalová, Magdaléna Janošíková, Romana Ulbrichtová, Viera Švihrová, Henrieta Hudečková, Eliška Sovová, Milan Sova, Jiří Vévoda

**Affiliations:** 1 Department of Occupational Medicine, University Hospital Olomouc and Faculty of Medicine and Dentistry, Palacký University Olomouc, I. P. Pavlova 6, 779 00 Olomouc, Czech Republic; 2 Department of Public Health, Jessenius Faculty of Medicine in Martin, Comenius University in Bratislava, Mala Hora 11149/4B, 036 01 Martin, Slovakia; 3 Department of Exercise Medicine and Cardiovascular Rehabilitation, University Hospital Olomouc and Faculty of Medicine and Dentistry, Palacký University Olomouc, 779 00 Olomouc, Czech Republic; 4 Department of Pulmonary Diseases and Tuberculosis, University Hospital Brno and Faculty of Medicine, Masaryk University, 625 00 Brno, Czech Republic; 5 Department of Humanities and Social Sciences, Faculty of Health Sciences, Palacký University in Olomouc, Hněvotínská 976/3, 775 15 Olomouc, Czech Republic

**Keywords:** burnout, healthcare worker, COVID-19, pandemic, emotional exhaustion, depersonalization, personal accomplishment

## Abstract

COVID-19 has led to an unprecedented strain on healthcare workers (HCWs). This study aimed to determine the prevalence of burnout in hospital employees during a prolonged pandemic-induced burden on healthcare systems. An online survey among employees of a Czech and Slovak university hospital was conducted between November 2021 and January 2022, approximately when the incidence rates peaked in both countries. The Maslach Burnout Inventory—Human Services Survey was applied. We obtained 807 completed questionnaires (75.1% from Czech employees, 91.2% from HCWs, 76.2% from women; mean age of 42.1 ± 11 years). Burnout in emotional exhaustion (EE) was found in 53.2%, depersonalization (DP) in 33%, and personal accomplishment (PA) in 47.8% of respondents. In total, 148 (18.3%) participants showed burnout in all dimensions, 184 (22.8%) in two, and 269 (33.3%) in at least one dimension. Burnout in EE and DP (65% and 43.7%) prevailed in physicians compared to other HCWs (48.6% and 28.8%). Respondents from COVID-19-dedicated units achieved burnout in the EE and DP dimensions with higher rates than non-frontline HCWs (58.1% and 40.9% vs. 49.9% and 27.7%). Almost two years of the previous overloading of healthcare services, caused by the COVID-19 pandemic, resulted in the relatively high prevalence of burnout in HCWs, especially in physicians and frontline HCWs.

## 1. Introduction

Burnout is a psychological syndrome emerging as a prolonged response to chronic stressors on the job. It is included in the 11th Revision of the International Classification of Diseases (ICD-11) as an occupational phenomenon [1,2]. The three key dimensions of this response are overwhelming emotional exhaustion (EE), feelings of detachment from the job known as depersonalization (DP), and a lack of personal accomplishment (PA). The exhaustion dimension is also described as wearing out, loss of energy, and fatigue. DP in human services occupations or, more generally, cynicism, is described as negative or inappropriate attitudes towards clients, irritability, loss of idealism, and withdrawal. Reduced PA is a sense of ineffectiveness accompanied by reduced productivity or capability, low morale, and an inability to cope. The significance of this three-dimensional model is that it clearly places the individual stress experience within a social context and involves the person’s conception of both self and others [1]. Burnout results from chronic workplace stress and increased work demand that has not been successfully managed [3,4]. It is characterized by energy depletion, negativity related to one’s job, and reduced professional efficacy. Previous research has linked burnout to various personal and patient care impacts, such as decreased professionalism, empathy, patient safety, teamwork failure, and increased medical errors and attritions [3,5].

In the first year of the coronavirus disease 2019 (COVID-19) pandemic, the global prevalence of anxiety and depression increased by a massive 25% in the general population [6]. The pandemic led to an unprecedented strain on healthcare services globally. During the pandemic, healthcare systems faced rising demands and limited resources. Considerable changes in healthcare delivery necessarily took place. These included cessation of routine services, repurposing of clinical areas, redeployment of staff to unfamiliar clinical environments, and in some circumstances, the rationing of services [7]. The very high number of cases and deaths, and the uncertainty about future COVID-19 waves raised awareness of these challenging working conditions and the need to address burnout by identifying possible solutions [8]. From the very beginning of the pandemic, it was thus assumed that burnout among healthcare workers (HCWs) would increase even more than in the general population [9]. Accordingly, studies from the first months of the pandemic demonstrated an increase in the prevalence of burnout in HCWs [10,11].

In the Czech Republic, there were five waves of a significant increase in incidence over the winter periods from the first cases in March 2020 until January 2022. During these waves, the weekly incidence of new cases exceeded 115 per 100,000 citizens with a maximum of over 300 new cases at the end of January 2022 [12,13]. In Slovakia, the epidemiological development of the pandemic was very similar, mostly with slightly lower incidence rates during the COVID-19 waves [14]. By the end of January 2022, there had been 348 deaths from COVID-19 per 100,000 citizens in the Czech Republic, and 327 in Slovakia. The beginning of 2022 represented the peak of the pandemic with the highest incidence of new cases so far, but in March 2022 there was a significant and long-lasting decrease in the incidence [12,13,14].

Various measures of burnout have been proposed so far. However, the Maslach Burnout Inventory General Survey (MBI) is the most frequently used. The MBI was specifically designed to assess the three dimensions of the burnout experience which had emerged from the earlier qualitative research. It has been considered the standard tool for research in this field. MBI has been translated and validated in many languages and adjusted for various kinds of professions [1,15].

The impact of long-term working conditions modified by the COVID-19 pandemic on the psychological well-being of HCWs is not yet well understood [7]. Therefore, the study aimed to determine the prevalence of burnout syndrome and its dimensions in university hospital employees, mostly HCWs, at a time of prolonged increase in the demand on the healthcare system due to the COVID-19 pandemic.

## 2. Materials and Methods

### 2.1. Study Population

The sample of the cross-sectional online survey consisted of all employees of University Hospital Olomouc (UHO, N = 4553) and Martin University Hospital (MUH, N = 2318). In early November 2021, the employees were asked to fill in an anonymous questionnaire distributed to individual hospital e-mail addresses accompanied by a cover letter providing basic information about the research purpose, ethics approval, and the email addresses and telephone numbers of relevant persons. Both the cover letter and the questionnaire were written in Czech for UHO and Slovak for MUH. A banner was also placed on the intranet of university hospitals calling for participation. Employees were instructed not to complete the questionnaire more than once. Data collection ended on 31 January 2022. In total, 807 completed questionnaires (of which 75.1% were from UHO employees) were obtained from 615 (76.2%) women and 192 (23.8%) men with a mean age of 42.1 ± 11 (median 42) years. In total, 736 (91.2%) participants were HCWs.

### 2.2. Survey Questionnaire

The applied Google form questionnaire was divided into three parts. At first, mostly closed-ended questions on respondents’ personal information including sex, education, age, duration of practice both in healthcare and in the university hospital, job type (i.e., physician, non-physician HCW, and non-HCW), managerial job position, night shift work and, in HCWs only, the healthcare sector in the hospital (i.e., inpatient, outpatient, combined). The second part was the official Czech version of MBI—Human Services Survey (HSS), comprising 22 items grouped into 3 subscales according to 3 dimensions of burnout (a 9-item subscale for EE, a 5-item subscale for DE and an 8-item subscale for PA). The severity of each item was assessed on an 8-point ordinal scale, based on the intensity of feelings that the respondent usually experienced from zero (none) to 7 (maximum), as one of the options for using the questionnaire proposed by its authors [16]. The third part included two closed-ended questions on the respondents who had already been infected with COVID-19, and the previous or current work at units dedicated to COVID-19 patients.

### 2.3. Data Analysis

The collected data were statistically analysed in the SPSS software (version 22.0). The continuous variables were presented as mean ± standard deviation (SD) or median. The categorical variables were presented as numbers (%). The normality of the data distribution was detected using the Shapiro–Wilk test. The differences between variables were compared using the Student’s *t*-test for variables with normal distribution or the Mann–Whitney U-test for variables with non-normal distribution, or the chi-squared test for categorical variables. Spearman’s correlation coefficients were determined between numerical variables and burnout scores. Cronbach’s alpha (α) was calculated to assess the reliability of measures and the internal consistency. A value ≥0.90 was considered as excellent reliability, ≤0.90–0.70 as high reliability, ≤0.70–0.50 as moderate reliability, and ≤0.50 as low reliability [17].

The MBI–HSS scores on each dimension were assessed separately; the three scores were not combined into a global score and each subscale had distinct thresholds. Based on recommended thresholds, each respondent reached a low, moderate, or high level of burnout for every dimension (Table 1) [16]. Higher scores on the EE and DP subscales indicated a higher burnout symptom burden; lower scores on the PA subscale indicated a higher burnout symptom burden [18]. A high level of any dimension was considered as presence of burnout [16].

## 3. Results

### 3.1. Characteristics of the Study Population

In the entire sample (*n* = 807), respondents had worked in health care for 19.5 ± 12.3 (median 20) years on average and their practice in university hospitals lasted 15.7 ± 11.8 (median 14) years (Table 2). A total of 331 (41%) participants had been infected with COVID-19. The two participating hospitals did not differ statistically significantly in these characteristics. However, significant differences between UHO and MUH respondents were recorded in the presence of the highest degree of education, job type, the healthcare sector in the hospital, the share of managerial job positions, and work at units dedicated to COVID-19 patients (Table 2). Respondents from MUH were significantly more often university-educated (78.6% vs. 68.2%, *p* = 0.005), physicians (50.2% vs. 26.7%, *p* < 0.001), inpatient workers (44, 3% vs. 38.3%, *p* < 0.001), more often reported the performance of a managerial position (22.9% vs. 17.2%, *p* = 0.041), and worked at COVID-19 dedicated units (45.8% vs. 37.6%, *p* < 0.025). The response rate was higher in UHO than MUH (17.7% vs. 8.7%, *p* < 0.001).

### 3.2. Burnout Syndrome in the Entire Sample

**Figure 1:**
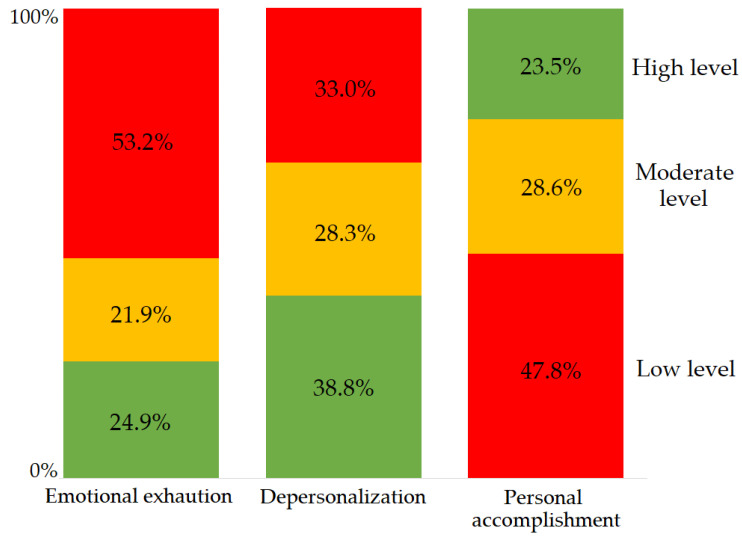
Distribution of achieved levels of burnout in all dimensions in the entire study sample (burnout depicted in red according to the above Data Analysis section).

In the entire sample, burnout in the EE dimension was found in 53.2%, DP in 33%, and PA in 47.8% of respondents, and on the contrary, a low level of burnout was recorded in 24.9%, 38.8% and 23.5% of respondents in the same order of the burnout dimensions (Table 2, Figure 1). In total, 148 (18.3%) participants showed burnout in all dimensions, 184 (22.8%) in two, and 269 (33.3%) in at least one dimension. More than half the share of the high level of burnout in the EE dimension was reflected in its mean score exceeding the burnout threshold. In the other two dimensions, the mean scores reflected a medium level of burnout. Both in the representation of the dimensions’ levels and their mean scores, UHO and MUH differed statistically significantly in EE and PA, with a high level of EE prevailing in the Slovak workplace (69.2% vs. 47.9%, *p* < 0.001) and a lack of PA in the Czech workplace (52% vs. 35.3%, *p* < 0.001; Table 2). In the entire sample, the reliability of the MBI–HSS was found to be high or even excellent: For all 22 items together, α = 0.799; separately for EE, α = 0.921; for DP, α = 0.770; for PA, α = 0.788.

### 3.3. Burnout Syndrome in Subgroups

There was a statistically significant difference in the prevalence of the DP dimension between women and men, with a high level of burnout in 29.1% of women and 45.3% of men (*p* < 0.001). Women and men were represented in nearly inverse proportions in a low level of DP (Table 3). The achieved score in the DP dimension correlated very weakly and inversely with age (r = −0.15, *p* < 0.001), duration of practice in healthcare (r = −0.17, *p* < 0.001), and in the university hospital (r = −0.13, *p* < 0.001). No other statistically significant correlations were found. The highest degree of education determined differences in the EE and DP dimensions. University-educated respondents achieved higher average scores and a high level of both dimensions was more frequent in them (Table 3). The identical situation in terms of the predominance of the EE and DP dimensions appeared in physicians compared to other HCWs and in HCWs compared to non-HCWs (Table 4). Respondents performing night work also achieved higher rates of EE and DP (Table 3). The managerial job position in university hospitals played a role only in the lack of PA, while individuals with managerial positions showed significantly less frequent burnout in this dimension (38.7% vs. 49.9%, *p* < 0.001; Table 3). There were no differences in burnout prevalence between subgroups of HCWs working in outpatient and inpatient care.

**Figure 2:**
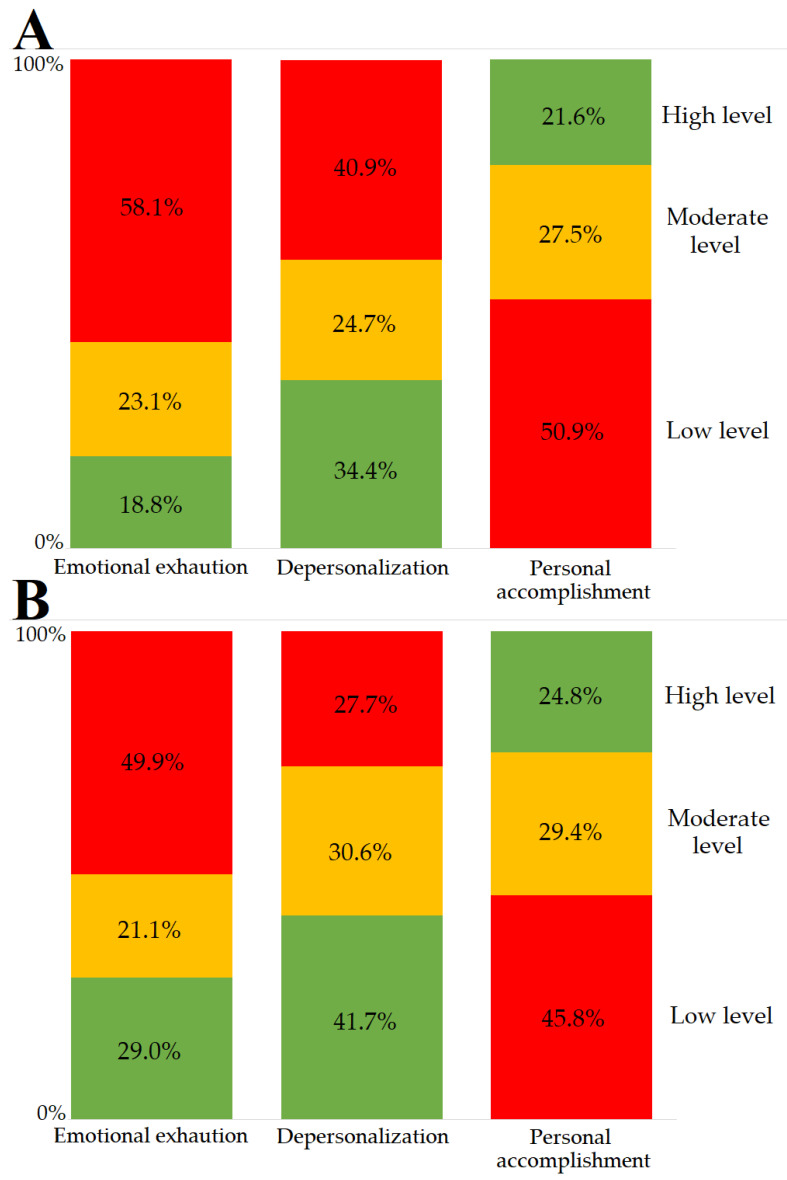
Comparison of the distribution of burnout levels in all dimensions in healthcare workers with the experience of the work at COVID-19 units (**A**) and those without the experience (**B**) (burnout depicted in red according to the above Data Analysis section).

Respondents with the experience of working at COVID-19-dedicated units more often and with higher mean scores exceeded in EE and DP burnout subscales over their non-frontline colleagues (Table 5, Figure 2). Burnout in the EE dimension was achieved by 58.1% of respondents working at COVID-19-dedicated units, and 49.9% of those who did not work at the units (*p* = 0.006). In the DP dimension, burnout occurred in 40.9% and 27.7% of participants in the same subgroups (*p* = 0.002). Those who had been infected with COVID-19 only showed a statistically higher mean score of PA compared to individuals who had not been infected with COVID-19.

## 4. Discussion

The results indicated a relatively high prevalence of burnout in both tertiary care hospitals. Three-quarters of respondents showed burnout in at least one of its dimensions. The burnout dimensions appeared with the following prevalence: EE, 53%; PA, 47.8%; and DP, 33%. This prevalence is close to the findings of a study by Orrù et al. conducted among HCWs from 45 different countries during the ongoing COVID-19 pandemic, in which EE was present in 56% and DP in 48.9% of respondents [19]. Although this occurrence is high, some studies had already demonstrated a considerable prevalence of burnout in HCWs before the pandemic’s onset. In a review study by Rothenberger, based on a search of the literature between 2000 and 2016, the prevalence of at least one dimension of burnout in US medical students, physicians in training, and practising physicians exceeded 50% [20]. Shanafelt et al., in their study among US physicians, showed an increase in burnout with 54.4% of the physicians reporting at least one symptom of burnout in 2014 compared with 45.5% in 2011 [21]. A systematic review and meta-analysis of 30 studies by Ghahramani et al. presented a comprehensive picture of the prevalence of burnout among HCWs during the COVID-19 pandemic. The pooled overall prevalence of burnout was 52% [95% confidence interval (CI) 40–63%]. EE, DP, and lack of PA were present in 51% (95% CI 42–61%), 52% (95% CI 39–65%), and 28% (95% CI 25–31%), respectively, which is very close to our results. Besides others, Ghahramani et al. pointed to significant regional differences and gradients in the prevalence of total burnout [22]. We noted a difference in the prevalence of EE and lack of PA between workplaces providing the same type of healthcare in two neighbouring countries, although the epidemiological situation in them had not differed during the pandemic. Finally, in their systematic review of 11 studies conducted during the COVID-19 pandemic, Gualano et al. stated that the prevalence of overall burnout ranged from 49.3% to 58% among HCWs [23].

Thus, it is evident that since the beginning of the COVID-19 pandemic, many studies on the prevalence of burnout and systematic reviews arising from them were conducted, however, given the turbulent evolution of the pandemic, they are all valuable evidence of a different stage of the pandemic documenting various circumstances. The questionnaire survey of the present work was carried out after almost two years of the pandemic, which was followed by a fundamental reduction in the rate of hospitalizations and mortality from COVID-19, together with a shift away from restrictive measures. In other words, soon after the survey was carried out, the situation normalized with a significant reduction in the burden on HCWs. The study thus demonstrates the prevalence of burnout syndrome at the time of the culmination of the pandemic-induced stress in HCWs. Being exposed to COVID-19 cases in hospitals, being quarantined, and experiencing huge performance pressure accompanied by a sudden surge of overwork, as well as frustration from failure to give optimal patient care, all negatively impact the mental well-being of HCWs. As the world headed into other years of the pandemic, these stressors became persistent and indefinite, heightening HCWs’ risk of burnout [24,25,26,27].

Various groups of HCWs seem to be differently prone to burnout. Several recent studies performed among HCWs during the COVID-19 pandemic identified a difference between sexes, such as Torrente et al., with women feeling more burnout than men (60.8% vs. 48.5%, *p* = 0.016) [28]. Similarly, but with an even greater prevalence in women, Ferry et al. found the same difference between the sexes (92% vs. 82%, *p*  =  0.004) [29]. Additionally, Di Giuseppe et al. identified female sex to be a predictor of perceived stress during the pandemic [30]. In our study, however, burnout in the DP dimension appeared significantly more frequently with higher scores in men. All three cited studies also showed that younger age was associated with a high risk of burnout syndrome, not specifically for its dimensions [28,29,30]. In the present work, lower age was also associated, albeit weakly, with a higher score of DP.

In our study, both university-educated respondents and physicians more often achieved scores above the burnout cut-offs in both EE and DP than other HCWs. Lasalvia et al. in their study among HCWs from a tertiary care hospital revealed that being a nurse increased the risk of burnout in the EE dimension in comparison to physicians (odds ratio 1.75; 95% CI 1.23 to 2.49), but not in the other dimensions [31]. On the other hand, Ruiz-Fernández et al. observed worse burnout score outcomes in physicians compared to nurses. They stated that physicians faced major ethical and moral decisions during the COVID-19 pandemic and unprecedented challenges for which they were most likely unprepared. The decisions included caring for seriously ill patients with limited or inadequate resources and having to prioritize some cases over others due to a widespread lack of resources [32]. The significantly higher share of physicians among Slovak respondents may have caused a higher prevalence of burnout in the EE dimension in the Slovak hospital.

More studies conducted during the pandemic objectified a greater burnout burden in frontline HCWs than in the non-frontline [28,31,33,34,35]. A systematic review of 44 studies by Sanghera et al. concluded that direct exposure to COVID-19 patients was the most common risk factor identified for all mental health outcomes including occupational burnout. Frontline HCWs and fewer years of working experience reported the worst outcomes [36]. Both characteristics worsened burnout in the present study too. The reasons for the worse psychological effects on frontline HCWs may include the following. Firstly, frontline workers had the most exposure risk and saw first-hand the effects of SARS-CoV-2 on patients. They may have feared being infected themselves and transmitting it to others. Secondly, personal protective equipment was often double-layered and uncomfortable. They must have been worn by staff for several hours without eating, drinking, or using the toilet. Many were becoming dehydrated from excessive sweating and developed skin conditions from excessive hand cleaning. Thirdly, due to the high morbidity and mortality associated with the disease, in addition to the reported unpredictable nature of deterioration, medical workers experienced feelings of helplessness [36]. Although it seems that the burden of the pandemic on the health sector has already decreased, in case of its re-intensification or any other situation imposing similarly strong and long-term demands, these reasons should be targeted and mitigated in time.

The systematic review by Ghahramani et al. focused on the prevalence of burnout dimensions by comparing subgroups of frontline and non-frontline HCWs. In their meta-analysis, the non-frontline group reported a high level of EE at 68% (95% CI 64–71%), while frontline exposure was associated with the highest level of DP at 57% (95% CI 35–78%) and a lack of PA at 29% (95% CI 17–41%) [22]. Workplace stress, time constraints, and anxiety stemming from staff shortages due to colleagues’ illness or redeployment to COVID-19-dedicated units may explain the high level of EE in non-frontline HCWs. A small number of available studies among non-frontline HCWs was emphasized by the authors [22]. Our study focused on both subgroups and revealed that frontline workers experiencing previous or current work at units dedicated to COVID-19 patients reported both higher prevalence and scores of EE and DP. This was the same finding as in the case of the study by Di Giuseppe et al., in which frontline workers reported higher scores of EE and DP compared to colleagues working at units not directly caring for patients with COVID-19 [30]. A systematic review by Maresca et al. supposed that the appropriate coping strategies employed by the team could be useful also in the prevention of psychological suffering, especially in contexts where working conditions were stressful. The application of these strategies (e.g., relaxation activities in the workplace and in free time) should be encouraged in HCWs by both employers and governments [37].

Future studies may show whether the decrease in the incidence of new cases of COVID-19 and the relief from the burden on healthcare systems lead to a decline in the prevalence of burnout among HCWs. All levels of healthcare should be studied and affected populations addressed, as burnout concerned not only tertiary care but also other levels including primary care during the pandemic [38]. It is important to continue to map and study burnout syndrome even in the context of post-COVID-19 conditions, with their highly variable symptoms including various psychological deteriorations, brain fog, etc. [39,40]. These conditions are prevalent, especially among HCWs [41].

Given the response rate of only around 12%, the study may be limited by selection bias. The survey took place during the culminating pandemic; filling out the questionnaire could have posed another unnecessary burden to HCWs. It is disputable whether a potential presence of burnout in an employee could have inclined them to fill in the questionnaire or oppositely not to take part in the survey, thus distorting the results. The ability to compare the results obtained from similar studies is limited by the region-dependent differences in the course of the COVID-19 pandemic and differences in healthcare settings. The use of only one tool for the burnout assessment is a limitation of the study. The use of an eight-point ordinal scale, instead of the more common seven-point scale, may also limit the comparison of our results. However, besides mean scores, all findings were also described in the form of relative levels (low, moderate, high) of all subscales, which are fully comparable when using different scales. On the other hand, a strength of the study is the survey conduction in two healthcare facilities providing the same type of care in neighbouring countries.

## 5. Conclusions

The results indicated a relatively high prevalence of burnout among HCWs in tertiary care hospitals from two culturally close Central European countries with similar epidemiological courses of the COVID-19 pandemic. The most common dimension of burnout was EE, followed by PA and DP. Three-quarters of respondents showed burnout in at least one of its dimensions, of which 18.3% reported in all three dimensions, and conversely, only a quarter showed no indication of burnout. Burnout represented a great concern for healthcare employees working in large tertiary-care hospitals during the COVID-19 pandemic, and its impact was more burdensome for physicians and HCWs compared to non-HCWs, younger employees, and HCWs working night shifts. Frontline HCWs demonstrated worse mental health outcomes in terms of higher EE and DP. The results reflected a situation after almost two years of the pandemic duration that created enormous demands on healthcare systems. Given the obtained results and those from literature sources, attention should be paid to addressing the high prevalence of burnout in HCWs, not only in the frontline and during the culmination of pandemics.

## Figures and Tables

**Table 1:** Score assessment of burnout in three dimensions [16].

Burnout Dimension/Subscale	Score	Interpretation
Emotional Exhaustion		
Low level	0–16	
Moderate level	17–26	
High level	27 and more	=burnout
Depersonalization		
Low level	0–6	
Moderate level	7–12	
High level	13 and more	=burnout
Personal accomplishment		
Low level	0–31	=burnout
Moderate level	32–38	
High level	39 and more	

**Table 2:** Characteristics of the study population.

Characteristics	Entire Sample	UHO	MUH	*p*-Value
Women (n, %)	615 (76.2%)	457 (75.4%)	158 (78.6%)	0.357
Men (n, %)	192 (23.8%)	149 (24.6%)	43 (21.4%)	
Age (years; mean ± SD)	42.1 ± 11	42.1 ± 10.7	42.3 ± 11.7	0.881
Duration of practice in healthcare (years; mean ± SD)	19.5 ± 12.3	19.6 ± 12.1	19.1 ± 13.1	0.499
Duration of practice in university hospital (years; mean ± SD)	15.7 ± 11.8	15.2 ± 11.5	17 ± 12.6	0.123
Education				
Secondary (n, %)	236 (29.2%)	193 (31.8%)	43 (21.4%)	0.005
University (n, %)	571 (70.8%)	413 (68.2%)	158 (78.6%)	
Job type in university hospital				
Physician (n, %)	263 (32.6%)	162 (26.7%)	101 (50.2%)	<0.001
Non-physician HCW (n, %)	473 (58.6%)	375 (61.9%)	98 (48.8%)	
Non-HCW (n, %)	71 (8.8%)	69 (11.4%)	2 (1%)	
Healthcare sector in the hospital				
Outpatient (n, %)	146 (18.1%)	126 (20.8%)	20 (10%)	<0.001
Inpatient (n, %)	321 (39.8%)	232 (38.3%)	89 (44.3%)	
Combined (n, %)	264 (32.7%)	172 (28.4%)	92 (45.8%)	
Managerial job position				
Yes (n, %)	150 (18.6%)	104 (17.2%)	46 (22.9%)	0.041
No (n, %)	657 (81.4%)	502 (82.8%)	155 (77.1%)	
Night work				
Yes (n, %)	510 (63.2%)	374 (61.7%)	136 (67.7%)	0.062
No (n, %)	297 (36.8%)	232 (38.3%)	65 (32.3%)	
Undergone COVID-19				
Yes (n, %)	331 (41%)	241 (39.8%)	90 (44.8%)	0.211
No (n, %)	476 (59%)	365 (60.2%)	111 (55.2%)	
Work at COVID-19 dedicated units				
Yes (n, %)	320 (39.7%)	228 (37.6%)	92 (45.8%)	0.025
Ne (n, %)	487 (60.3%)	378 (62.4%)	109 (54.2%)	
Burnout				
Score EE (mean ± SD)	28.31 ± 13,44	26.74 ± 14.99	33.05 ± 13.04	<0.001
Score DP (mean ± SD)	9.81 ± 8,49	9.77 ± 7.09	9.93 ± 7.8	0.912
Score PA (mean ± SD)	31.73 ± 13,44	30.6 ± 9.3	35.14 ± 8.85	<0.001
Prevalence of EE				
Low level (n, %)	201 (24.9%)	176 (29%)	25 (12.4%)	<0.001
Moderate level (n, %)	177 (21.9%)	140 (23.1%)	37 (18.4%)	
High level (n, %)	429 (53.2%)	290 (47.9%)	139 (69.2%)	
Prevalence of DP				
Low level (n, %)	313 (38.8%)	232 (38.3%)	81 (40.3%)	0.362
Moderate level (n, %)	228 (28.3%)	179 (29.5%)	49 (24.4%)	
High level (n, %)	266 (33%)	195 (32.2%)	71 (35.3%)	
Prevalence of PA				
Low level (n, %)	386 (47.8%)	315 (52%)	71 (35.3%)	<0.001
Moderate level (n, %)	231 (28.6%)	178 (29.4%)	53 (26.4%)	
High level (n, %)	190 (23.5%)	113 (18.6%)	77 (38.3%)	

SD, standard deviation; UHO, University hospital Olomouc; MUH, Martin university Hospital; HCWs, healthcare worker; EE, emotional exhaustion; DP, depersonalization; PA, personal accomplishment.

**Table 3:** Burnout dimensions with respect to respondents’ subgroups.

Burnout	Sex			Education			Night Work			Managerial Job Position		
	Women (*n* = 615)	Men (*n* = 192)	*p*-Value	Secondary (*n* = 236)	University (*n* = 571)	*p*-Value	Yes (*n* = 510)	No (*n* = 297)	*p*-Value	Yes (*n* = 150)	No (*n* = 657)	*p*-Value
Burnout dimensions (mean score)												
Score EE	28.05 ± 14.57	29.17 ± 15.42	0.330	25.76 ± 13.98	29.37 ± 14.98	0.002	29.47 ± 14.77	26.33 ± 14.6	0.004	27.71 ± 14.48	28.45 ± 14.85	0.643
Score DP	9.14 ± 7.09	11.97 ± 7.44	< 0.001	8.58 ± 6.53	10.32 ± 7.5	0.004	10.83 ± 7.4	8.07 ± 6.7	< 0.001	8.81 ± 6.84	10.04 ± 7.35	0.068
Score PA	31.83 ± 9.26	31.42 ± 9.82	0.712	31.64 ± 9.61	31.78 ± 9.31	0.997	31.75 ± 9.11	31.7 ± 9.89	0.931	33.73 ± 10.3	31.28 ± 9.13	0.003
Prevalence of EE												
Low level	158 (25.7%)	43 (22.4%)	0.498	70 (29.7%)	131 (22.9%)	0.045	113 (22.2%)	88 (29.6%)	0.011	36 (24%)	165 (25.1%)	0.669
Moderate level	137 (22.3%)	40 (20.8%)		56 (23.7%)	121 (21.2%)		106 (20.8%)	71 (23.9%)		37 (24.7%)	140 (21.3%)	
High level	320 (52%)	109 (56.8%)		110 (46.6%)	319 (55.9%)		291 (57.1%)	138 (46.5%)		77 (51.3%)	352 (53.6%)	
Prevalence of DP												
Low level	265 (43.1%)	48 (25%)	< 0.001	105 (44.5%)	208 (36.4%)	0.033	172 (33.7%)	141 (47.5%)	< 0.001	66 (44%)	247 (37.6%)	0.085
Moderate level	171 (27.8%)	57 (29.7%)		68 (28.8%)	160 (28%)		142 (27.8%)	86 (29%)		46 (30.7%)	182 (27.7%)	
High level	179 (29.1%)	87 (45.3%)		63 (26.7%)	203 (35.6%)		196 (38.4%)	70 (23.6%)		38 (25.3%)	228 (34.7%)	
Prevalence of PA												
Low level	297 (48.3%)	89 (46.4%)	0.132	113 (47.9%)	273 (47.8%)	0.864	245 (48%)	141 (47.5%)	0.938	58 (38.7%)	328 (49.9%)	< 0.001
Moderate level	183 (29.8%)	48 (25%)		70 (29.7%)	161 (28.2%)		147 (28.8%)	84 (28.3%)		39 (26%)	192 (29.2%)	
High level	135 (22%)	55 (28.6%)		53 (22.5%)	137 (24%)		118 (23.1%)	72 (24.2%)		53 (35.3%)	137 (20.9%)	

EE, emotional exhaustion; DP, depersonalization; PA, personal accomplishment.

**Table 4:** Burnout dimensions with respect to job type in hospital.

Burnout	Job type in University Hospital					
	Physicians (*n* = 263)	Non-Physician HCWs (*n* = 473)	*p*-Value	HCWs (*n* = 736)	Non-HCWs (*n* = 71)	*p*-Value
Burnout dimensions (mean score)						
Score EE	32.16 ± 14.51	26.97 ± 14.43	<0.001	28.82 ± 14.66	23 ± 15	0.002
Score DP	11.52 ± 7.75	9.2 ± 6.84	<0.001	10.03 ± 7.26	7.6 ± 7	0.003
Score PA	31.49 ± 9.36	32.2 ± 9.3	0.254	31.95 ± 9.32	29.5 ± 9.9	0.068
Prevalence of EE						
Low level	39 (14.8%)	133 (28.1%)	<0.001	172 (23.4%)	29 (40.8%)	0.004
Moderate level	53 (20.2%)	110 (23.3%)		163 (22.1%)	14 (19.7%)	
High level	171 (65%)	230 (48.6%)		401 (54.5%)	28 (39.4%)	
Prevalence of DP						
Low level	80 (30.4%)	196 (41.4%)	<0.001	276 (37.5%)	37 (52.1%)	0.031
Moderate level	68 (25.9%)	141 (29.8%)		209 (28.4%)	19 (26.8%)	
High level	115 (43.7%)	136 (28.8%)		251 (34.1%)	15 (21.1%)	
Prevalence of PA						
Low level	132 (50.2%)	219 (46.3%)	0.504	351 (47.7%)	35 (49.3%)	0.094
Moderate level	67 (25.5%)	138 (29.2%)		205 (27.9%)	26 (36.6%)	
High level	64 (24.3%)	116 (24.5%)		180 (24.5%)	10 (14.1%)	

HCW, healthcare worker; EE, emotional exhaustion; DP, depersonalization; PA, personal accomplishment.

**Table 5:** Burnout dimensions with respect to work at units dedicated to COVID-19 patients and undergone COVID-19.

Burnout	Work at COVID-19 Dedicated Units			Undergone COVID-19		
	Yes (*n* = 320)	No (*n* = 487)	*p*-Value	Yes (*n* = 331)	No (*n* = 476)	*p*-Value
Burnout dimensions (mean score)						
Score EE	30.45 ± 14.56	26.91 ± 14.76	0.0014	28.81 ± 14.59	27.97 ± 14.91	0.473
Score DP	11.13 ± 7.44	8.95 ± 7.03	<0.001	9.67 ± 7.08	9.91 ± 7.4	0.789
Score PA	31.27 ± 9.42	32.04 ± 9.37	0.222	32.56 ± 9.54	31.16 ± 9.26	0.029
Prevalence of EE						
Low level	60 (18.8%)	141 (29%)	0.006	75 (22.7%)	126 (26.5%)	0.457
Moderate level	74 (23.1%)	103 (21.1%)		76 (23%)	101 (21.2%)	
High level	186 (58.1%)	243 (49.9%)		180 (54.4%)	249 (52.3%)	
Prevalence of DP						
Low level	110 (34.4%)	203 (41.7%)	0.002	128 (38.7%)	185 (38.9%)	0.511
Moderate level	79 (24.7%)	149 (30.6%)		100 (30.2%)	128 (26.9%)	
High level	131 (40.9%)	135 (27.7%)		103 (31.1%)	163 (34.2%)	
Prevalence of PA						
Low level	163 (50.9%)	223 (45.8%)	0.327	145 (43.8%)	241 (50.6%)	0.134
Moderate level	88 (27.5%)	143 (29.4%)		99 (29.9%)	132 (27.7%)	
High level	69 (21.6%)	121 (24.8%)		87 (26.3%)	103 (21.6%)	

EE, emotional exhaustion; DP, depersonalization; PA, personal accomplishment.

## Data Availability

The data that support the findings of this study are available from the corresponding author, [J.V.], upon reasonable request.
